# Review of Platensimycin and Platencin: Inhibitors of β-Ketoacyl-acyl Carrier Protein (ACP) Synthase III (FabH)

**DOI:** 10.3390/molecules200916127

**Published:** 2015-09-03

**Authors:** Ruofeng Shang, Jianping Liang, Yunpeng Yi, Yu Liu, Jiatu Wang

**Affiliations:** 1 Key Laboratory of New Animal Drug Project of Gansu Province, Key Laboratory of Veterinary Pharmaceutical Development, Ministry of Agriculture, Lanzhou Institute of Husbandry and Pharmaceutical Sciences of CAAS, Lanzhou 730050, China; E-Mails: shangrf1974@163.com (R.S.); liangjianping@caas.cn (J.L.); yiyunpeng88@163.com (Y.Y.); yangguang8684@163.com (Y.L.); 2 Affiliated Hospital of Gansu University of Chinese Medicine, Lanzhou 730000, China

**Keywords:** platensimycin, platencin, drug resistance, antibacterial activities, synthesis, analogues

## Abstract

Platensimycin and platencin were successively discovered from the strain *Streptomyces platensis* through systematic screening. These natural products have been defined as promising agents for fighting multidrug resistance in bacteria by targeting type II fatty acid synthesis with slightly different mechanisms. Bioactivity studies have shown that platensimycin and platencin offer great potential to inhibit many resistant bacteria with no cross-resistance or toxicity observed *in vivo.* This review summarizes the general information on platensimycin and platencin, including antibacterial and self-resistant mechanisms. Furthermore, the total synthesis pathways of platensimycin and platencin and their analogues from recent studies are presented.

## 1. Introduction

**Figure 1:**
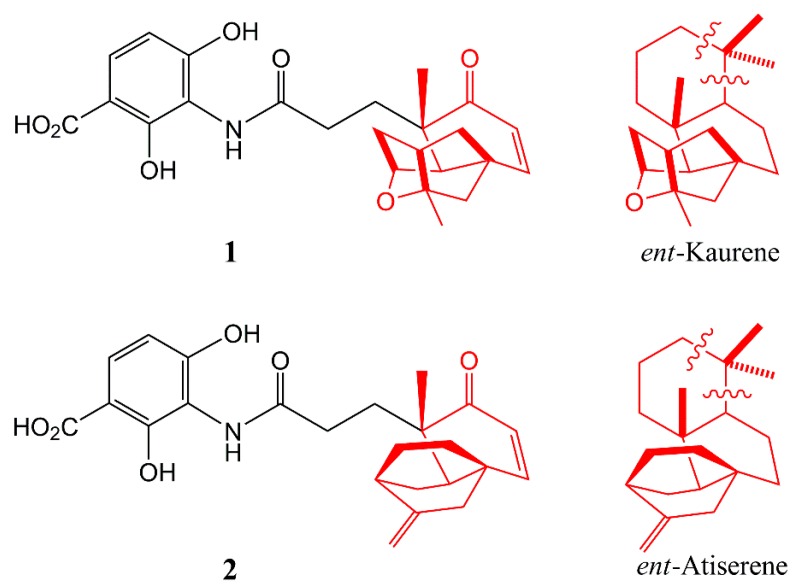
Structures of platensimycin **1** and platencin **2** and their biosynthetic relationship to *ent*-kaurene and *ent*-atiserene.

Many available drugs have reduced or lost their curative effect, leading to increased morbidity and mortality, because of the emergence and spread of multidrug resistant bacteria [1]. The drastic increase in pathogenic bacterial resistance, especially in multiresistant bacteria, is one of the most serious problems endangering human health and urgently needs for effective solutions [2,3]. Chemical modifications of existing scaffolds have afforded antibiotics with improved activity and have served well the development of new and effective antibiotics in past decades. However, such modifications are becoming increasingly challenging [4]. Therefore, the discovery of novel antibiotic chemical scaffolds with new modes of action to overcome drug resistance is crucial [5]. Natural products are an important tool for this approach, particularly platensimycin (**1**) and platencin (**2**) (Figure 1).

The natural products platensimycin and platencin, discovered by employing a novel antisense differential sensitivity screening strategy, were reported recently from soil bacterial strains of *Streptomyces platensis* [6,7,8]. Platensimycin inhibits fatty acid acyl carrier protein synthase II (FabF) selectively [6], whereas platencin is a balanced dual inhibitor of both FabF and fatty acid acyl carrier protein synthase III (FabH) [9]. Because of their unique antibacterial mechanism, these natural products show potent, broad-spectrum Gram-positive *in vitro* activity, including against antibiotic-resistant bacteria, such as methicillin-resistant *Staphylococcus aureus* (MRSA), vancomycin-intermediate *S. aureus* (VISA), and vancomycin-resistant *Enterococci* (VRE) [4]. Importantly, both platensimycin and platencin exhibit no cross-resistance to clinically relevant pathogens and show *in vivo* efficacy without toxicity [10].

This review will provide an overview of the isolation, antibacterial activities, biosynthetic machineries, and antibacterial and self-resistant mechanisms of platensimycin and platencin. Moreover, total synthesis and their analogues in recent years are also described.

## 2. Isolation, Antibacterial Activities, and Biosynthesis

Platensimycin was first discovered by the Merck research group through a systematic screening of approximately 250,000 natural product extracts as part of their target-based whole-cell screening strategy using an antisense differential sensitivity assay [11]. This product was isolated (2–4 mg/L) from the fermentation broth of *Streptomyces platensis* (MA7327 and MA7331) by a two-step process, a capture step followed by reversed-phase high performance liquid chromatography (HPLC), and the structure was elucidated by 2D nuclear magnetic resonance (NMR) methods and confirmed by X-ray crystallographic analysis [11]. Platensimycin was ultimately proven to be a new class of antibiotics with no cross-resistance in other classes of antibiotic-resistant bacteria [12,13]. It is a selective inhibitor for *S. aureus* FabF and *Escherichia coli* (*E. coli*) FabF/B enzymes with IC_50_ values of 48 and 160 nm, respectively [14]. *In vitro* antibacterial studies have shown that platensimycin is effective against Gram-positive bacteria with minimal inhibitory concentration (MIC) values of 0.1–0.32 mg/mL [15].

Another extract of a new strain of *Streptomyces platensis* (MA7339) was isolated from a soil sample and identified by the same research group after the discovery of platensimycin. Platencin was discovered and isolated by bioassay-guided fractionation of this extract [4,16]. Unlike platensimycin, platencin possesses a polycyclic enone skeleton bearing an exocyclic double bond instead of an ether linkage, and is a balanced dual inhibitor of both FabH (IC_50_ = 9.2 mm) and FabF (IC_50_ = 4.6 mm). Platencin shows broad-spectrum antibacterial activity against various bacterial strains, including drug resistant bacteria such as MRSA, linezolid-resistant *S. aureus*, vancomycin intermediate *S. aureus* and VRE with MIC values ≥0.06–4 mg/mL [14].

Feeding experiments using ^13^C precursors suggest that the aminobenzoic acid moieties of platensimycin and platencin were biosynthesized from pyruvate and acetate via the tricarboxylic acid cycle and phosphoenolpyruvate in the strains of *Streptomyces platensis*. The C-17 polycyclic enone acid moiety of platensimycin was biosynthesized via a non-mevalonate terpenoid pathway by the oxidative excision of three carbons from *ent*-kaurene (Figure 1) [17,18]. Recent studies showed that the biosynthesis of platencin in *Streptomyces platensis* MA7327 and MA7339 is controlled by *ent*-atiserene (Figure 1) synthases, a new pathway for diterpenoid biosynthesis that is different from *ent*-kaurene synthases [19].

## 3. Antibacterial and Self-Resistant Mechanism

**Figure 2:**
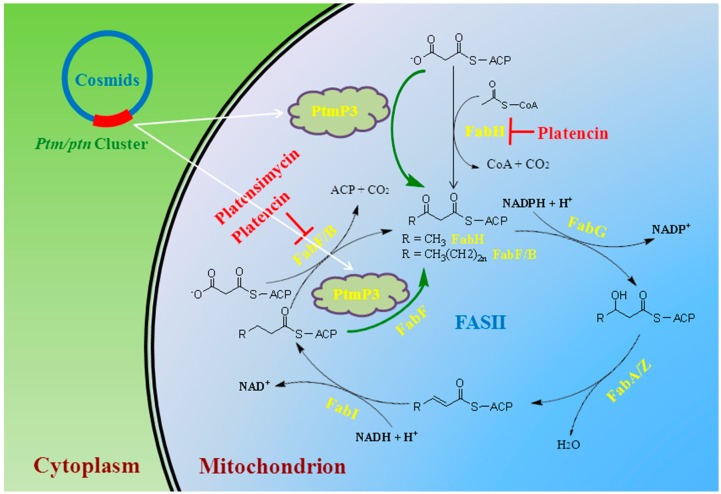
A diagram of the bacterial fatty acid synthesis cycle (FASII). Highlighted in red are (i) platensimycin inhibiting FabF/B and platencin dually inhibiting FabF and FabH, and (ii) the two complementary mechanisms of platensimycin and platencin resistance in *S. platensis* by target replacement and target modification. Adapted from Peterson *et al.* [10].

Fatty acids form the building blocks of many cellular structures and are required for energy storage in bacteria [9]. The synthesis of fatty acids in bacteria is conducted by type II bacterial fatty acid synthesis (FASII) which is different from the synthesis found in eukaryotes (mammals and fungi; type I). Type II FAS consists of many discrete enzymes- involved in condensation, reduction and dehydration [20]. Platensimycin and platencin interfere with the synthesis of fatty acids by selectively inhibiting FabF, an elongation-condensing enzyme whose main function is to add acetate units to the growing fatty acid chain [9]. A FabH/FabF PAGE elongation assay with the crude *S. aureus* cytosolic proteins, which closely mimics the events inside living cells, suggested that platensimycin is a selective FabF inhibitor, whereas platencin is a dual inhibitor with similar inhibition efficiency for both FabF and FabH (Figure 2) and thus inhibits fatty acid synthesis in *S. aureus* through a synergistic effect [4].

Pathogens have generally gained their resistance genes by horizontal gene transfer from non-pathogenic bacteria, with one potential source being antibiotic producing bacteria that developed highly effective mechanisms to avoid suicide [21]. Therefore, it is important to understand the self-resistance mechanisms within platensimycin and platencin producing organisms. A standard disk diffusion assay identified the *ptmP3* or *ptnP3* gene within the platensimycin or platencin biosynthetic cluster in the S. *platensis* MA7327 and MA7339 strains as the major resistance conferring element. The FabF gene within the housekeeping fatty acid synthase locus was identified as the second resistance conferring element. PtmP3/PtnP3 and FabF, therefore, confer platensimycin and platencin resistance by target replacement (*i.e.*, FabF and FabH by PtmP3) and target modification (*i.e.*, a platensimycin-insensitive variant of FabF), respectively (Figure 2). PtmP3/PtnP3 also represents an unprecedented mechanism for fatty acid biosynthesis in which FabH and FabF are functionally replaced by a single condensing enzyme (*i.e.*, a platensimycin-insensitive variant of FabF) [10].

## 4. Recent Total Synthesis

**Scheme 1:**
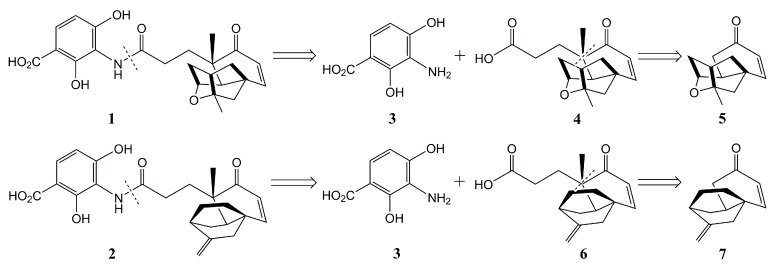
The retrosynthetic analysis of platensimycin **1** and platencin **2**.

Structural studies revealed that platensimycin and platencin consist of a compact aliphatic tetracyclic ketolide core and polycyclic enone core, respectively, that were connected through a propionate tether to a highly oxygenated aromatic ring [9,22]. Therefore, the syntheses of platensimycin or platencin are generally divided into two sub-targets: the aromatic amine **3** and the carboxylic acid **4** or **6** constructed by tetracyclic cage **5** or Nicolaou’s intermediate **7** are the key intermediates for synthesizing platensimycin or platencin, respectively (Scheme 1) [23]. Since the discovery of platensimycin and platencin, many concise synthetic routes targeting their complex molecular framework, especially the aliphatic tetracyclic ketolide core or polycyclic enone core, have been developed.

### 4.1. Zhu’s Total Syntheses of (±)-Platensimycin and Platencinusing a Cascade Cyclization Approach

**Scheme 2:**
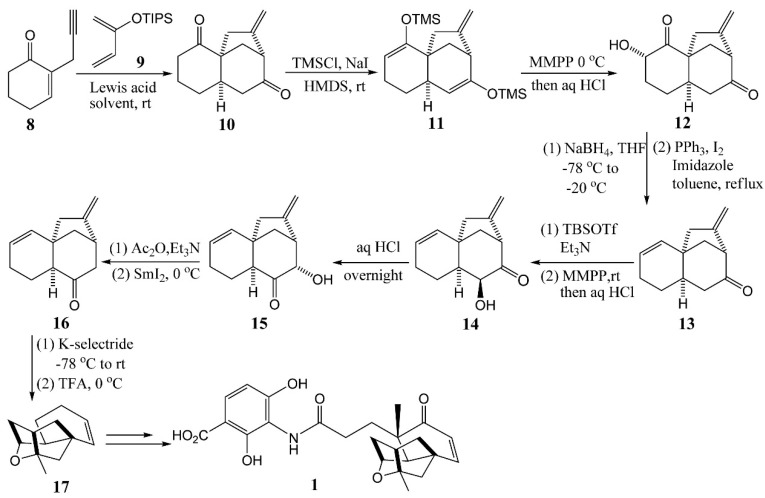
A bifunctional Lewis acid induced cascade cyclization to the synthesis of platensimycin.

Bifunctional Lewis acids are highly useful for developing cascade cyclization reactions because they can induce cyclization reactions via forming σ- and/or π-complexes with the substrates, as well as the intermediate(s) generated *in situ* [24]. In 2012, a Chinese research group offered a new route for the synthesis of platensimycin (Scheme 2) using a mild and efficient bifunctional Lewis acid-induced cascade cyclization reaction for rapid construction of the tricyclic core of *ent*-kaurenoids [25]. With ZnBr_2_ as the bifunctional Lewis acid, enone **8** and diene **9** underwent cascade cyclization smoothly at room temperature and provided the tricyclic diketone **10** in one pot with good yields (86%) and high diastereoselectivity. The resulting diketone **10** was converted to the di-tetramethylsilane (TMS) enol ether **11**. Subsequent epoxidation using magnesium monoperoxyphthalate provided R-hydroxy ketone **12** selectively in 65% yield. The reduction of the ketone moiety of **12** followed by elimination of the resulting diol afforded **13** in good yields. Silyl enol formation followed by magnesium monoperoxyphthalate (MMPP) epoxidation of **13** provided R-hydroxyl ketone **14** as a single diastereomer, which can be equilibrated to the more stable R-hydroxyl ketone **15**. Alcohol **15** was then acetylated and deacetoxylated using SmI_2_ to afford ketone **16**. Finally, the reduction of ketone **16** with K-selectride followed by trifluoroacetic acid treatment afforded the Snider intermediate **17** (32% overall yields in 11 steps), which could be converted to **1** according to the literature report [26,27].

**Scheme 3:**
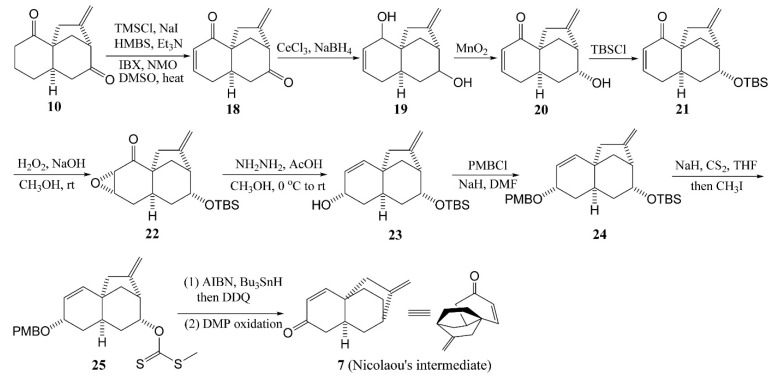
Lewis acid induced cascade cyclization to the synthesis of platencin.

Again using the Lewis acid induced cascade cyclization approach to platencin, with minor modifications (Scheme 3), Zhu *et al.* [28] synthesized the tricyclic diketone **10**, which was further converted to enone **18** by oxidation. Then, enone **18** was reduced using Luche reduction conditions to diol **19** in a single diastereomer. The allylic alcohol of **19** was thus selectively oxidized and protected with *t*-butyldimethylsilyl (TBS) to afford enone **21**, followed by stereoselective conversion to **23** via the Wharton transposition protocol. After protecting and functional group manipulations, compound **23** was converted to the bicycle octane **25** using Yoshimitsu’s procedures. Finally, oxidative removal of the *p*-methoxybenzyl (PMB) ether followed by oxidation of the resulting alcohol completed the synthesis of Nicolaou’s intermediate **7**, which can also be converted to platencin according to the literature procedures [29].

The two synthesis routes employed the same cyclized product **10** as the precursor for the formal synthesis of Snider or Nicolaou’s intermediates. However, the synthesis of Nicolaou’s intermediate was a relatively short route than that for Snider intermediate, and finally gave a good overall yield for platencin (38% overall yields in 11 steps).

### 4.2. Horii’s Synthesis of Tetracyclic Cage **5**

**Scheme 4:**
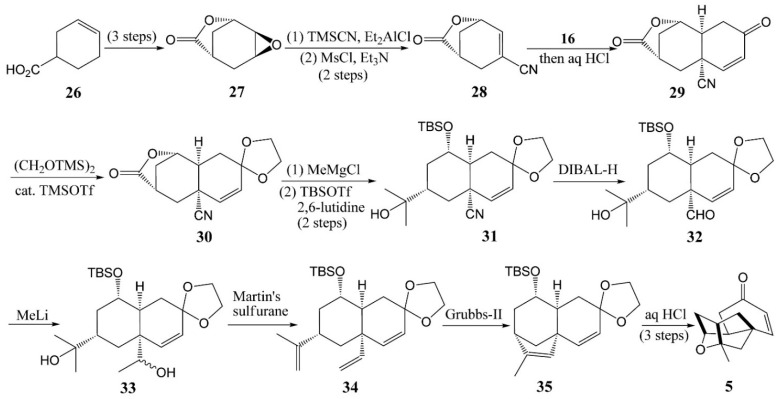
Stereoselective synthesis of the tetracyclic cage **5**.

To date, researchers have placed particular emphasis on the diverse approaches for chemical synthesis of the tetracyclic cage **5** because it is an important intermediate for approaching **1** [23]. As shown in Scheme 4 [30], epoxide **27** was readily obtained from **26** by a known three-step sequence, followed by regioselective trans-diaxial ring opening and dehydration of **27** to **28**. The Diels-Alder reaction of **28** with Rawal’s diene B gave **29** as a single isomer. The lactone **30**, which was obtained by protection of the ketone functionality of **29**, was treated with MeMgCl in tetrahydrofuran (THF)/hexamethylphosphoramide to give a diol intermediate, the secondary hydroxy group of which was then protected as its *t*-butyldi-methylsilyl ether **31**. The nitrile **31** was reduced with diisobutyl aluminum hydride to give aldehyde **32**, and the subsequent addition of MeLi to **32** afforded **33** as an inconsequential 1:1 mixture of diastereomers. The ring closing metathesis (RCM) precursor **34** obtained by subjecting the diol **23** to an excess amount of Martin’s sulfurane could be achieved efficiently by slowly adding the second-generation Grubbs catalyst in the presence of 1,4-benzoquinone at an elevated temperature to afford the tricyclic product **35**. Finally, treatment of **35** with hydrochloric acid to remove the silyl and acetal protecting groups induced concurrent intramolecular etherification, providing tetracyclic cage **5**. Although this was a new method for construction tricyclic diketone **29** as a single isomer, the preparation of the cyano lactone **28** inevitably prolonged the synthesis and gave a poor (6.6%) overall yield for the tetracyclic cage **5**.

### 4.3. Gamal’s Synthesis of Platencin

**Scheme 5:**
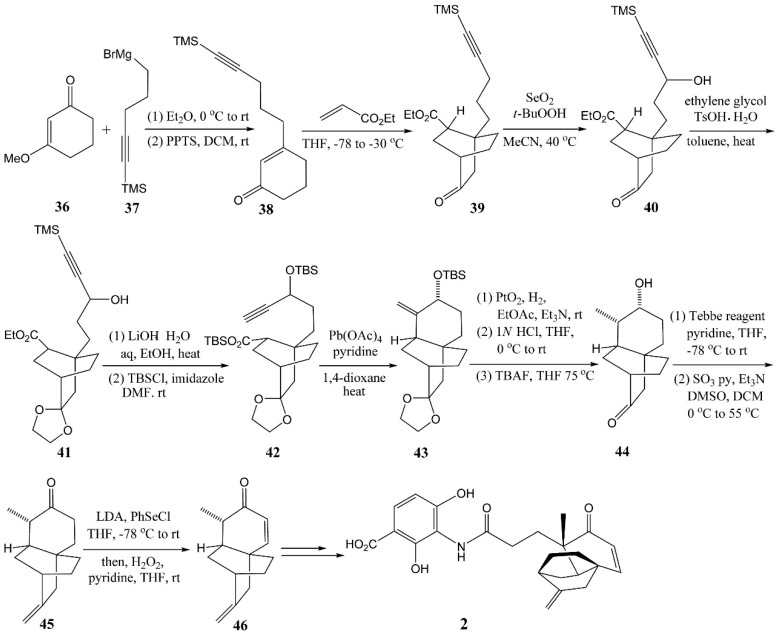
Synthesis of platencin via decarboxylative radical cyclization.

Gamal *et al.* established a new route to platencin via decarboxylative radical cyclization, shown in Scheme 5 [31]. Enone **38** was prepared by adding Grignard reagent **37** to enone **36**, followed by simple dehydration of the resultant tertiary alcohol. Then, enone **38** was converted to **39** under conventional sequential Michael reaction conditions. The introduction of oxygen functionality was conducted by SeO_2_-mediated propargylic oxidation leading to alcohol **40**. Ketalization of the carbonyl group of **40** under azeotropic dehydration conditions furnished ketal **41**, which was hydrolyzed with LiOH and further silylated with *t*-butyldi-methylsilyl chloride/imidazole to give the disilylated material **42**. The decarboxylative radical cyclization of **42** then produced the desired compound **43**. After hydrogenation with PtO_2_ in the presence of triethylamine, compound **43** was converted to a saturated product, and its ketal and the silyl ether were sequentially cleaved using 1 N HCl followed by tetrabutylammonium fluoride to provide hydroxyketone **44**. Hydroxyketone **44** was then subjected to Tebbe olefination followed by Parikh-Doering oxidation to produce ketone **45**. The conventional phenylselenylation of ketone **45** with lithium diisopropylamide/phenylselenyl chloride, followed by oxidative elimination with H_2_O_2_, successfully gave enone **46** which could be converted to platencin [29].

The decarboxylative radical cyclization of alkynyl silyl ester **42** was the key step for constructing the tricyclic core of platencin. With Pb(OAc)_4_ in the presence of pyridine in refluxing 1,4-dioxane, the key decarboxylation allowed the rapid construction of the twisted polycyclic compound **43**, but with a poor (30%) yeild.

### 4.4. Chang’s Synthesis of Platencin

**Scheme 6:**
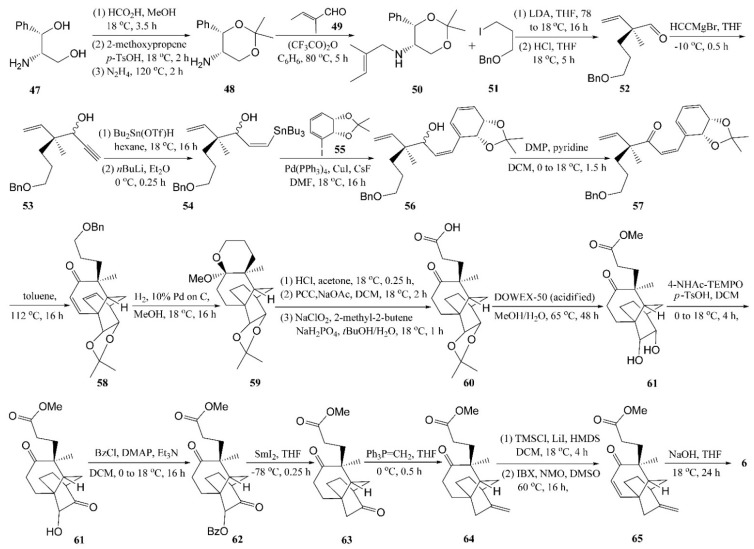
Synthesis the tricarbocyclic framework of platencin using an intramolecular Diels-Alder approach.

Chang *et al.* used an intramolecular Diels-Alder (IMDA) approach for the direct assembly of the tricarboxylic framework associated with platencin, outlined in Scheme 6 [32].

The commercially available amino-diol **47** was converted into 1,3-dioxane **48** under the conditions defined by Nordin and Thomas [33]. The condensation of **48** with aldehyde **49** in the presence of trifluoroacetic anhydride gave the imine **50**, which was deprotonated using lithium diisopropylamide. The resulting anion was alkylated with the benzyl ether **51**, followed by an acidic workup that gave the desired aldehyde **52**. Treating compound **52** with ethynylmagnesium bromide in THF gave the anticipated propargyl alcohol **53**. The sequential treatment of compound **53** with the readily prepared Lewis acidic hydrostannane Bu_2_Sn (OTf) H followed by *n*BuLi gave the required Z-configured alkene **54**. The tetraene **56** was obtained as an approximately 1:1 mixture of diastereoisomers by the treatment of an approximately 1:1 mixture of **54** and acetonide **55** with [Pd(PPh_3_)_4_] in the presence of CuI and CsF. Compound **56** was oxidized to the corresponding ketone **57** using the Dess-Martin periodinane in the presence of pyridine. Then, the anticipated intramolecular Diels-Alder adduct **58** was obtained from **57** under-reflux in toluene. Compound **58** was exposed to dihydrogen in the presence of 5% Pd to give the crystalline ketal **59**, which was oxidized with pyridinium chlorochromate to give carboxylic acid **60**. Cleavage of **60** gave the diol **61**, which could be regioselectively monooxidized using the sterically demanding oxammonium salt to afford acyloin **62**. After esterification, the acyloin **62** was converted to benzoate **63** and subsequently generated the diketo ester **64** through a samarium iodide-promoted reduction reaction. Subjecting compound **64** to a Wittig olefination reaction using Ph_3_P=CH_2_ gave the desired alkene **65**, which could finally be converted to **6** by the saponification of ester **65** using aqueous sodium hydroxide in THF.

Although the original total synthesis of platencin reported by Nicolaou and co-workers in 2008 [29] used the same IMDA approach to establish the framework **7** in an enantioselective manner, the pivotal aspect of this work was the use of the enantiomerically pure *cis*-1,2-dihydrocatechol (which is obtained by the whole-cell biotransformation of iodobenzene using a genetically engineered form of *E. coli*) as the starting material [32]. Furthermore, derivatives of enone **7** that already incorporated a suitably constituted quaternary carbon center adjacent to the carbonyl residue and thus were more readily elaborated to (−)-platencin, were generated by this modified route in as highly controlled a manner as possible.

## 5. Recent Analogues and Their Antibacterial Activities

Since the discovery of platensimycin and platencin, significant effort has been undertaken to prepare platensimycin and platencin analogues with high antibacterial activities by organic synthesis. Meanwhile, structure activity relationship (SAR) studies were performed to find more effective analogues with high efficiency. The SAR studies have revealed that modifications on the 2,4-dihydroxybenzoic acid moiety of platensimycin or platencin usually diminish most of the biological activity of this antibiotic [34,35,36]. In sharp contrast, decorations or modifications on the caged ring often retain good biological activity [34,37].

Because the tetracyclic/tricyclic core of platensimycin or platencin is relatively difficult to prepare chemically, several groups have focused their attention on the replacement of the “difficult-to-synthesize” moiety [38].

**Figure 3:**
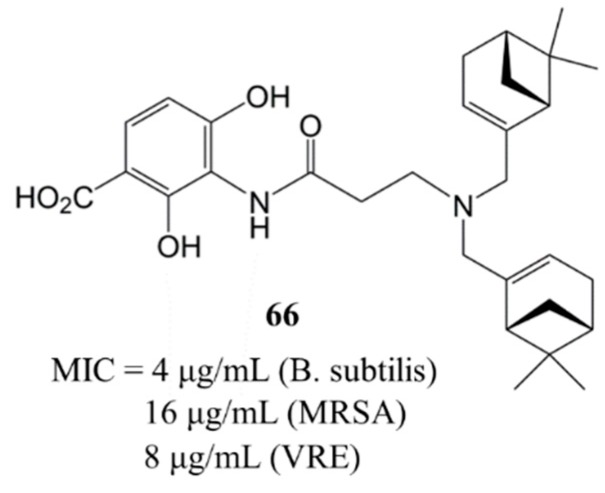
Structure and antibacterial activity (MIC, μg/mL) of compound **66**.

A combinatorial strategy in which dihydroxybenzoic acid was coupled to a variety of alkyl chains was developed by Wang *et al.* [38] based on the finding that FAS enzymes utilize substrates with long alkyl chains to make fatty acids and the idea that there ought to be a hydrophobic pocket at or near the active site of these enzymes. They designed and synthesized a series of 2,4-dihydroxybenzoic acid esters with a terminal amino group. From bioactivity studies, only compound **66** showed excellent *in vitro* antibacterial activity against *B. subtilis* (3160), MRSA and VRE, with MIC values of 4, 16 and 8 μg/mL, respectively (Figure 3).

Plesch’s group also focused on the development of novel simplified platensimycin analogues and thereby synthesized a series of compounds containing the anilide group and the ketone, both identified as essential for binding of the active components to the Fab F complex [39]. After a standardized agar diffusion assay, ketones **67** and **68** showed a broad spectrum of antibacterial activity, comparable to the antibiotics tetracycline and clotrimazol (Table 1).

**Table 1:** Agar diffusion assay for compounds **67**, **68**, tetracycline and clotrimazol (50 mg/disc).

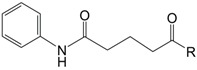

Strains	67: R = Ph	68: R = *Trans* H_3_C-CH=CH-	Tetracycline	Clotrimazol
*Escherichia coli*	11	10	25	0
*Pseudomonas antimicrobia*	14	10	23	0
*Staphylococcus equorum*	0	11	23	9
*Streptococcus entericus*	16	14	12	11
*Candida glabrata*	20	17	nt	15
*Aspergillus niger*	0	6	nt	15
*Yarrowia lipolytica*	0	7	nt	20
*Hyphopichia burtonii*	10	15	nt	17

Zones of inhibition (in mm); nt, not tested; 0, no measurable zone of inhibition.

Fisher *et al.* described an approach to platensimycin analogue design using structure-based ligand design (SBLD), a powerful approach that can facilitate the discovery of bioactive small molecules when high quality structural information is available [40]. They designed and synthesized a series of platensimycin analogous and determined the affinities of these compounds for the C163Q mutant of FabF using a WaterLOGSY [41] competition binding assay. The resulting designed compounds were shown to bind in the platensimycin binding site of the C163Q mutant of FabF, and, in several cases, compounds **69**, **70**, **71** and **72** had higher affinity (lower dissociation constants) than the reference compound **73** (Table 2).

**Table 2:** Dissociation constants of compounds **69**, **70**, **71**, **72** and reference compound **73** for the C163Q mutant of FabF.

Structure	R	Compounds	Kd/μM
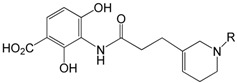	-SO_2_NHBOC	**69**	50 ± 10
	-SO_2_NH_2_	**70**	100 ± 20
	-CONH*i*Pr	**71**	110 ± 30
	3-pyridyl	**72**	120 ± 20
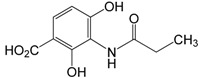		**73**	650 ± 90

## 6. Conclusions

Multidrug resistance has become a serious global concern. Research on new antibacterial drugs that are effective against resistant pathogenic bacteria is highly crucial. This article describes the efforts of chemists to overcome multidrug resistance in the case of the new antibiotic platensimycin isolated from extracts of *Streptomyces platensis*, and its analogue platencin. These natural products interfere with the synthesis of fatty acids by selectively inhibiting -FabF, and are of particular interest because they exhibit a new mode of action that overcomes existing drug resistance. Antibacterial studies have shown that these new antimicrobials have great potential in inhibiting MRSA, VRE, VISA and penicillin-resistant *Streptococcus pneumoniae*. The fascinating molecular architecture, novel mode of action and high antibacterial activities have inspired many different approaches to the synthesis and chemical modifications of platensimycin and platencin in a relatively short time.

## Abbreviation

FabF: Fatty acid acyl carrier protein synthases II
FabH: Fatty acid acyl carrier protein synthases III
MRSA: Methicillin-resistant *Staphylococcus aureus*
VISA: Vancomycin-intermediate *S. aureus*
VRE: Vancomycin-resistant *Enterococci*
HPLC: High performance liquid chromatography
NMR: Nuclear magnetic resonance
*E. coli*: *Escherichia coli*
MIC: Minimal inhibitory concentration
FASII: Type II bacterial fatty acid synthesis
TMS: Tetramethylsilane
MMPP: Magnesium monoperoxyphthalate
TBS: *t*-Butyldimethylsilyl
PMB: *p*-Methoxybenzyl
THF: Tetrahydrofuran
RCM: Ring closing metathesis
IMDA: Intramolecular Diels-Alder
SAR: Structure activity relationship
